# Selection of the Optimal Herbal Compositions of Red Clover and Pomegranate According to Their Protective Effect against Climacteric Symptoms in Ovariectomized Mice

**DOI:** 10.3390/nu8080447

**Published:** 2016-07-23

**Authors:** Su Jin Kang, Beom Rak Choi, Seung Hee Kim, Hae Yeon Yi, Hye Rim Park, Chang Hyun Song, Sae Kwang Ku, Young Joon Lee

**Affiliations:** 1 The Medical Research Center for Globalization of Herbal Medicine, Daegu Haany University, 1, Haanydaero, Gyeongsan, Gyeongsangbuk-Do 38610, Korea; vegonia1@hanmail.net (S.J.K.); dvmsong@hotmail.com (C.H.S.); 2 Department of Preventive medicine, College of Korean Medicine, Daegu Haany University, 1, Haanydaero, Gyeongsan, Gyeongsangbuk-Do 38610, Korea; 3 Research Institute, Health-Love Co., Ltd., Anyang 13946, Korea; brchoi@health-love.com (B.R.C.); key7413@health-love.com (S.H.K.); Leehaeyun@health-love.com (H.Y.Y.); hrpark@health-love.com (H.R.P.); 4 Department of Histology and Anatomy, College of Korean Medicine, Daegu Haany University, 1, Haanydaero, Gyeongsan, Gyeongsangbuk-Do 38610, Korea

**Keywords:** dried pomegranate concentrate powder, red clover dry extracts, anti-climacteric effects, ovariectomy

## Abstract

This study aimed to ascertain the optimal range of red clover dry extracts (RC) and dried pomegranate concentrate powder (PCP) to induce anti-climacteric effects. Thus, the dose ranges showing protective effect of mixed formulae consisting of RC and PCP were examined in ovariectomized mice. At 28 days after bilateral ovariectomy (OVX), mixed herbal compositions (RC:PCP = 1:1, 1:2, 1:4, 1:8, 2:1, 4:1, and 8:1) were administered orally, at 120 mg/kg once daily for 84 days. We evaluated that RC and PCP mixture attenuate OVX-caused obesity, hyperlipidemia, hepatic steatosis, and osteoporosis. Compared to OVX-induced control mice, body weight and abdominal fat weight in OVX-induced mice were significantly decreased, concomitantly with increase of uterus weight by RC:PCP mixture. Additionally, significant increases in serum estradiol levels were observed in all RC:PCP-treated mice. RC:PCP mixture also showed protective effect against OVX-induced hyperlipidemia, hepatic steatosis. Total body and femur mean bone mineral density (BMD), osteocalcin, bALP contents were effectively increased by RC:PCP mixture. Taken together, RC:PCP mixture (2:1, 1:1, and 4:1) has remarkable protective effects against the changes induced by OVX. In particular, RC:PCP mixture (2:1) shows the strongest effect and may be considered as a potential protective agent against climacteric symptoms.

## 1. Introduction

During the climacteric period, many women between the ages of 40 and 65 years experience and complain of uncomfortable symptoms such as vasomotor symptoms, night sweats, cognitive impairment, insomnia, depression, and irritability [1,2]. In addition, body weight gain, fatigue and hot flashes are major symptoms characterized of menopause women. Many symptoms of menopause were closely associated with level of estrogen. These postmenopausal conditions are related to an increased risk of metabolic diseases, such as obesity, heart disease, diabetes, and hypertension, due to estrogen deficiency [3,4]. Additionally, estrogen deficiency is recognized as a common risk factor for osteoporosis. Estrogen deficiency is related to an atherogenic lipid profile, characterized by high density lipoprotein (HDL)-cholesterol, low density lipoprotein (LDL)-cholesterol, triglyceride levels [5], central adiposity [6], increased diastolic pressure [7], and increased insulin resistance [8]. Thus, for attenuating estrogen deficiency of menopausal symptoms, synthetic estrogens were administered to women during menopause. These hormones are as protective as estrogens in the reduction of symptoms [9,10,11].

As mentioned above, hormone therapy has been used to protect women against climacteric symptoms; however, long-term exposure can cause cardiovascular events and breast cancer [9,10,11]. Thus, phytosubstances from plants, as an alternative to hormone therapy, have attracted much attention [12,13,14]. They have similar structures to estrogens and can bind to estrogen receptors, and therefore were named phytoestrogens (PEs). Several PEs, plant-derived chemicals, act as free radical scavengers. Isoflavones, derived from soy and soy derivatives, are representative PEs. Genistein and daidzein are the most abundant and well studied. This class of PEs can also be found in clover. Purified phytohormones show improved activity in the body and enhanced bioavailability [12]. PEs can bind to estrogen receptors due to the presence of a phenolic ring and function like estrogens [12,13,15]. Coumestrol and the isoflavonoids genistein, daidzein, and their plant precursors are found mainly in soybeans and clover [16]. Isoflavones, especially those derived from plants, have various biological activities, can improve metabolic symptoms [17], and have bone-protective effects [18] during menopause. Recently, it has been suggested that various pharmacological effects of plant extracts can be enhanced synergistically by appropriate mixed formulations [19,20,21].

Red clover (RC, *Trifolium pratense* L.) shows estrogenic effects due to isoflavones and, to a lesser extent, coumestans [22]. Thus, RC botanical dietary supplements have been used for the treatment of menopausal symptoms, maintenance/improvement of cardiovascular health, and their reported benign effects on the breast, endometrium, and neural structures [23]. The isoflavonoids formononetin, biochanin A, genistein, and daidzain are present in RC as glycosides and malonates [24].

Pomegranates (*Punica granatum* L.) contain various flavonoids and anthocyanidins, and their main active substances are polyphenols, which show antioxidative, antimutagenic, antiinflammatory, and antimicrobial activities [25]. The phytoestrogenic effects of pomegranate [26] are due mainly to isoflavonoids via antioxidant and antiinflammatory pathways [15,27]. Recently, pomegranate extract has been shown to be a selective estrogen receptor modulator [28].

Based on the information above, we hypothesized that dried pomegranate concentrate powder (PCP) might potentiate the anti-climacteric effects of RC. Appropriate mixed formulations of RC and PCP may be expected to show more favorable synergistic anti-climacteric effects. Thus, this study aimed to ascertain the optimal range of RC + PCP to induce anti-climacteric effects. Additionally, we examined whether the anti-climacteric activity of RC could be enhanced by the addition of PCP in ovariectomized (OVX) mice. 

## 2. Experimental Section

### 2.1. Animals and Husbandry

Kwl:ddY mice (virgin female specific pathogen-free outbred mice) (6 weeks old upon receipt) (Kiwa, Wakayama, Japan) were selected follow to the acclimatization for 16 days. The animals (4 mice per polycarbonate cage) were maintained with a controlled temperature (20–25 °C) and humidity (45%–55%) under 12-h:12-h light:dark cycles. Tap water and normal rodents pellet diet (Table 1; 38057; Purinafeed, Seungnam, Korea) were given ad libitum. All mice were placed in individual cages containing 150 g of diet and 250 mL of water and the remaining amounts were measured at 24 h after feed supply using an automatic electronic balance (Precisa Instruments) and a measuring cylinder (Pyrex, Corning, NY, USA), respectively. This was regarded as the individual daily food (g/24 h/mouse) and water (mL/24 h/mouse) rations [29]. All laboratory animals were controlled according to national regulations for the usage and welfare of laboratory animals and approved by the Institutional Animal Care and Use Committee of Daegu Haany University (Gyeongsan, Gyeongbuk, Korea) prior to the experiments (Approval No. DHU2014-020). In addition, experiments on osteoporosis were performed based on United States Food and Drug Administration guidelines [30].

### 2.2. Experimental Groups

The dose level of 120 mg/kg was selected as dosages of mixed formula consisted of RC:PCP on the clinical dosage in human (mouse dosage = about 12-fold that of human dosage; (600 mg/60 kg) × 12 = 120 mg/kg). The experimental groups were divided into the following 12 groups (8 mice per group): sham vehicle control (sham control); OVX-operated (OVX control); OVX-operated mice + 17β-estradiol (0.03 μg/head); OVX-operated mice + 120 mg/kg RC; OVX-operated mice + 120 mg/kg PCP; OVX-operated mice + 120 mg/kg RC:PCP 1:1 (60:60 mg/kg) mixture; OVX-operated mice + 120 mg/kg RC:PCP 1:2 (40:80 mg/kg) mixture; OVX-operated mice + 120 mg/kg RC:PCP 1:4 (24:96 mg/kg) mixture; OVX-operated mice + 120 mg/kg RC:PCP 1:8 (13:107 mg/kg) mixture; OVX-operated mice + 120 mg/kg RC:PCP 2:1 (80:40 mg/kg) mixture; OVX-operated mice + 120 mg/kg RC:PCP 4:1 (96:24 mg/kg) mixture; OVX-operated mice + 120 mg/kg RC:PCP 8:1 (107:13 mg/kg) (g/g) mixture. 

### 2.3. Experimental Design

In the present study, Kwl:ddY mice (6 weeks old upon receipt) were prepared and ovariectomy was performed 16 days after acclimatization. Mice were anesthetized with an intraperitoneal injection of 25 mg/kg of Zoletile (Zoletile 50™; Virbac Laboratories, Carros, France) and maintained with 1%–1.5% isoflurane (Hana Pharmaceutical Co., Hwasung, Korea) in a mixture of 70% N_2_O and 28.5% O_2_. Surgery was conducted according to established methods [31]. The second group of mice underwent a sham operation in which a similar incision in the linea alba was made but bilateral ovariectomy was not performed.

**Figure 1:**
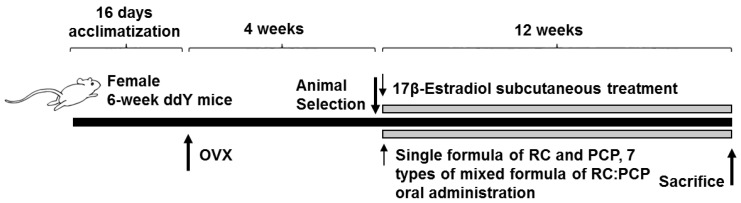
Experimental designs used in this study. OVX = Bilateral ovariectomy, RC = Red clover dry extracts, PCP = Pomegranate Concentrate Powder, RC:PCP = Mixed formulations consisted of RC and PCP (g/g).

At 28 days after surgery, 8 mice per group were selected based on body weight and RC (120 mg/kg), PCP (120 mg/kg), or an RC:PCP mixture (g/g) was administered orally once a day for 84 days. Standardized RC, PCP, and RC:PCP 1:1, 1:2, 1:4, 1:8, 2:1, 4:1, and 8:1 (g/g) mixture were provided by HEALTH-LOVE Co., Ltd. (Anyang, Korea). The RC substance contained 8% total isoflavones, 0.62% genistein, 5.43% biochanin A, 3.66% formononetin, and 0.47% daidzein suspended in 12 mg/mL of distilled water. The PCP substance contained 0.90 mg/g of ellagic acid dissolved in 12 mg/mL of distilled water. Appropriate amounts of RC, PCP, and the RC:PCP mixture (g/g) were directly suspended or dissolved in distilled water and administered in a volume of 10 mL/kg. In OVX and sham control mice, only distilled water was administered orally as a vehicle in equal volumes and periods instead of the herbal formulas. In addition, 17β-estradiol (Sigma-Aldrich, St. Louis, MO, USA) 0.03 μg was dissolved in 0.2 mL of sterilized saline, and subcutaneously treated on the dorsal back skins in a volume of 0.2 mL/mouse (0.03 μg/head/day). After 84 days of continuous oral administration, the mice were anesthetized with 50 mg/kg of Zoletile and dissected according to established methods [32] (Figure 1). 

Differences in body weight were measured at the time of ovariectomy, 1 day before RC, PCP, and RC:PCP administration, and once a week from the initiation of administration to termination using an automatic electronic balance (Precisa Instruments, Dietikon, Switzerland) [29]. At ovariectomy, the first administration, and at termination, food, but not water, was withheld from the experimental animals (approximately 18 h prior) to reduce differences due to feeding. 

### 2.4. Measurement of the Bone Mineral Density (BMD) and Body Fat Density

The mean BMD of total body and the right femur were determined using in live dual-energy X-ray absorptionmetry (DEXA; InAlyzer, Medikors, Seungnam, Korea). In addition, mean fat densities on the body and abdominal cavity regions of each mouse, respectively. 

### 2.5. Organ Weight Measurements

At sacrifice, the abdominal fat pads deposited in the abdominal cavity, total liver, and uterus (including vagina) were collected after removing the surrounding connective tissues, muscles, and any debris, after which the weights of organs were measured at g levels for absolute wet-weights. To reduce the individual body weight differences, the relative weights (% of body weight) were calculated at sacrifice. 

### 2.6. Bone Weight Measurements

At sacrifice, the right sides of the femurs were collected after removing the surrounding connective tissues, muscles, and any debris. The bone weight was measured at g levels regarding absolute wet-weights, and they were dried at 120 °C for 8 h in a high temperature dry oven (LDO-080N, Daihan Labtech Co., Seoul, Korea) for measurements of dry bone weights. Next, dried bones were carbonized at 800 °C for 6 h in a furnace (LEF-1055-1, Daihan Labtech Co.) to measure ash absolute weights. To reduce the individual body weight differences, the relative weight (%) was calculated the proportion of absolute wet/dry/ash weight based on the body weight at sacrifice. 

### 2.7. Measurement of Bone Strengths

Bone strength was detected as failure load (FL). FL of mid-shaft regions of right femurs were detected using a three-point bending test to failure using a computerized testing machine (SV-H1000, Japan Instrumentation System Co., Tokyo, Japan) as N (Newton).

### 2.8. Blood Collection

For serum biochemistry, approximately 1 mL of whole blood was collected from vena cava at sacrifice and separated from the serum by centrifugation at 21,000× *g* for 10 min at 4 °C using a clotting activated serum tube. All serum samples were frozen at −150 °C until they were assayed.

### 2.9. Serum Biochemistry

Serum aminotransferase (AST), alanine aminotransferase (ALT), total cholesterol (TC), LDL, and triglyceride (TG) levels were detected using an automated blood analyzer (Hemagen Analyst; Hemagen Diagnostic, Columbia, MD, USA), and HDL levels were measured using another typed automated blood analyzer (AU400; Olympus, Tokyo, Japan). In addition, serum osteocalcin levels were detected using a Mouse Osteocalcin ELISA Kit (Immutopics, San Clemente, CA, USA) as ng/mL levels, and serum bALP levels were detected using the Mouse bALP ELISA kit (Quidel Corp., San Diego, CA, USA), as U/L levels as pg/mL, with an ELISA Reader (Tecan, Männedorf, Switzerland). In addition, serum estradiol contents were measured using the chemiluminescent immunoassay technique (ECLIA, Roche e411 immunoassay analyzer, Roche, Penzberg, Germany) from the separated serum at sacrifice in all mice.

### 2.10. Abdominal Fat Pads, Uterus, and Liver Histological Procedures 

Sampled tissues were fixed in 10% neutral buffered formalin (NBF). After paraffin embedding, 3–4 μm serial sections were prepared. Representative sections were stained with hematoxylin and eosin (H&E) for light microscopic examination. Alternatively, portions of liver that had been dehydrated in 30% sucrose solutions were sectioned by cryostat for staining the lipids with oil red [33]. The total thicknesses of abdominal fat pads were measured using an automated image analysis processor (*i*Solution FL; ver. 9.1, IMT *i*-solution Inc., Quebec, QC, Canada) as mm/mouse, and mean diameters of dorsal abdominal white adipocytes were calculated in restricted view fields on a computer monitor, using an automated image analysis processor, as μm. At least 10 white adipocytes per fat pad were considered for histomorphometrical analysis according to our previously established methods [29,33,34]. In addition, total full, mucosa, and epithelial thicknesses of the uterus (μm/uterus) were detected as percentages of uterine glands located in the mucosa (%/mucosa of uterus) using an automated image analyzer. To observe steatosis in the liver, the percentage of fatty change regions in hepatic parenchyma was calculated as percentages between 1 field of liver (%/mm^2^ of hepatic parenchyma) under oil red staining, and mean diameters of hepatocytes were calculated in restricted view fields on a computer monitor under H&E staining using an automated image analysis processor, as μm; at least 10 hepatocytes per liver were considered. 

### 2.11. Bone Histological Procedures

The left sides of each mouse femur were separated and fixed in 10% NBF, after which they were decalcified in decalcifying solution (24.4% formic acid and 0.5 N sodium hydroxide) for 3 days (mixed decalcifying solution was exchanged once a day for 3 days). The samples were then embedded in paraffin, sectioned (3~4 μm), and stained with Safranin-O stain. In addition, bone histomorphometry was conducted using an automated image analyzer under microscopy (Nikon, Tokyo, Japan) to examine bone mass and structure with bone resorption in a uniform area of epiphyseal or cortical bone regions of the femur (growth plate regions were excluded). Cortical bone thickness was also measured in the mid-shaft regions of the femur. Trabecular bone volume (TV/BV, TBV; %), thickness of trabecular bone (Tbt; μm/trabecular bone), number (Tbn; mean numbers of trabecular bone/epiphyseal regions), length (Tbl; mm/trabecular bone), and cortical bone thickness (Cbt; μm/mid-shaft cortical bone) were measured for bone mass and structure, and osteoclast cell number (Ocn; mean osteoclast cell numbers/epiphyseal regions) and ratio (OS/BS; %) were measured for bone resorption as described previously [20,31,35]. 

### 2.12. Statistical Analyses

All values for the eight mice in this experiment were expressed as means ± SD. Multiple comparison tests were performed a two-tailed test for the different dose groups. Variance homogeneity was examined using the Levene test. If the Levene test indicated no significant deviations from variance homogeneity, the data were analyzed using the one-way ANOVA test followed by the least-significant differences test to determine which group comparisons were significantly different. When significant deviations from variance homogeneity were observed on the Levene test, the non-parametric the Kruskal-Wallis test was conducted. When a significant difference was observed on the Kruskal-Wallis test, the Mann-Whitney U test was conducted to determine the specific pairs of groups that were significantly different. Statistical analyses were conducted using the SPSS for Windows software package (ver. 14.0; SPSS Inc., Chicago, IL, USA).

## 3. Results

### 3.1. Body Weight and Gains

Body weights were the higher in all OVX induced mice than in shame control mice, with significant increases in body weight gains during the 4-week OVX recovery/induction periods. In contrast, OVX-induced body weight increase was significantly prevented by all seven RC:PCP mixed formulae, from 14, 21, and 28 days after the initial treatment, compared with OVX control mice. Especially, the mice treated with 2:1, 1:1, and 4:1. 

Mixtures had a lower body weight increase than that treated with RC or PCP alone on days 28, 42, and 49 after the initial treatment, in that order (Table 2, Appendix A).

### 3.2. Food Consumption

Compared with sham control mice at 1, 3, 7, 28, 56, and 83 days after initial administration, OVX-induced mice showed significant increases in food consumption. No significant change in daily food consumption was observed in the RC alone, PCP alone, or any of the seven RC:PCP mixed formula-treated mice, compared with OVX control mice (Appendix B).

### 3.3. Organ Weights

OVX-induced mice showed significant higher in relative abdominal fat pad weight deposited in the abdominal cavity compared with sham control mice. In contrast, uterus and liver relative weights were lower in OVX-induced mice than in sham control mice. However, these changes in abdominal fat, uterus and liver weights were prevented by estradiol, RC, PCP and RC:PCP treatments (Table 3). The mean relative weights of abdominal fat pads deposited into the abdominal cavity in OVX controls were changed by 1491.59%, compared with the sham control, and by −78.86%, −41.76%, −36.56%, −65.51%, −74.73%, and −62.83%, in the estradiol-, RC alone-, PCP alone-, and 1:1, 2:1, and 4:1 RC:PCP mixed formula-treated mice, respectively, compared with the OVX controls. The mean relative uterine weights of OVX were changed by −92.53%, compared with sham controls, and by 372.56%, 74.57%, 67.51%, 145.55%, 200.64%, and 142.46% in estradiol-, RC alone-, PCP alone-, and 1:1, 2:1, and 4:1 RC:PCP mixed formula-treated mice, respectively, compared with OVX controls.

### 3.4. Changes in Abdominal Fat Pad, Uterus, and Liver Histopathology

**Figure 2:**
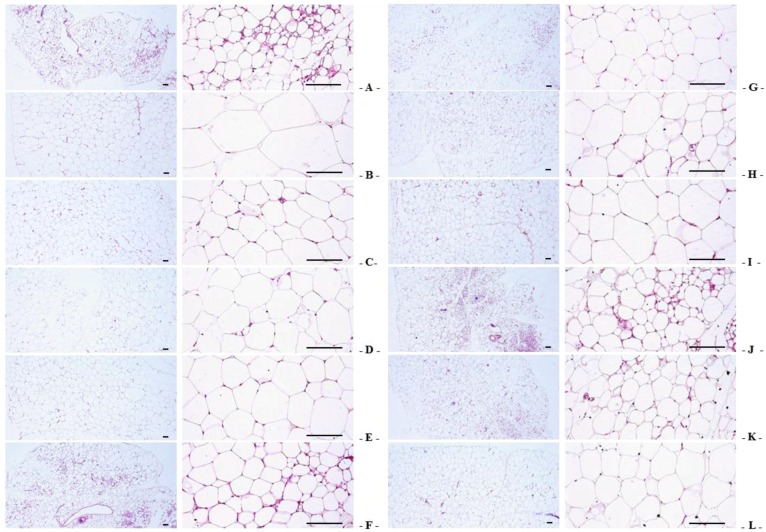
Representative histological images of the adipocytes, taken from sham-operated or OVX ddY mice abdominal fat pads deposited in abdominal cavity. (**A**) Sham vehicle control mice; (**B**) OVX control mice; (**C**) 17β-estradiol OVX induced mice; (**D**) RC alone administered OVX induced mice; (**E**) PCP alone administered OVX induced mice; (**F**–**L**) RC:PCP 1:1, 1:2, 1:4, 1:8, 2:1, 4:1, and 8:1 mixtures administered OVX-induced mice, respectively. OVX = Ovariectomy; RC = Red clover dry extracts; PCP = Pomegranate Concentrate Powder; RC:PCP = Mixed formulations consisted of RC and PCP (g/g). All H&E stain. Scale bars = 120 μm.

Significant increases in the thickness of abdominal fat pads deposited into the abdominal cavity and the mean adipocyte diameters were observed in OVX-induced mice due to the deposition in adipose tissues in the abdominal cavity and the hypertrophy of adipocytes, respectively. However, the thickness of abdominal fat pads and their mean diameters of adipocytes in all test substance-administrated mice (including estradiol treated mice) were the lower than in OVX control mice. Especially, RC:PCP 2:1, 1:1 and 4:1 mixed formula treated mice showed more inhibitory activities in the deposition in adipose tissues and the hypertrophy of adipocytes than compared with RC- or PCP-treated mice, in that orders, respectively (Table 4, Figure 2). 

**Figure 3:**
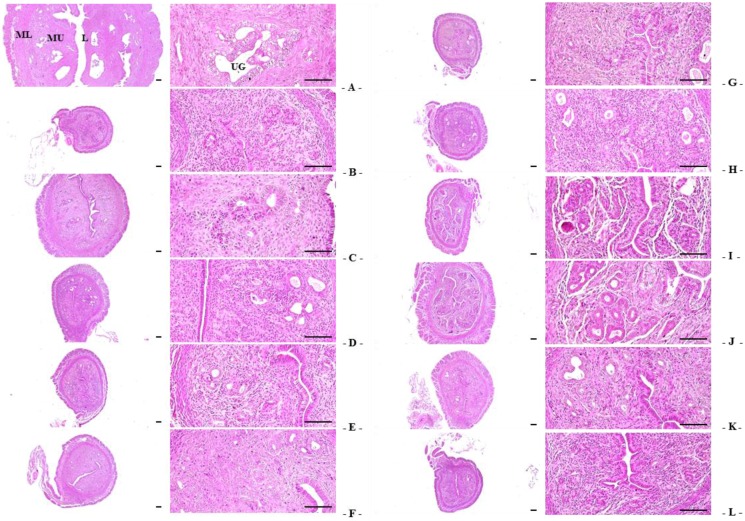
Representative histological images of the left uterus horn, taken from sham-operated or OVX ddY mice. (**A**) Sham vehicle control mice; (**B**) OVX control mice; (**C**) 17β-estradiol OVX induced mice; (**D**) RC alone administered OVX induced mice; (**E**) PCP alone administered OVX induced mice; (**F**–**L**) RC:PCP 1:1, 1:2, 1:4, 1:8, 2:1, 4:1, and 8:1 mixtures administered OVX-induced mice, respectively. OVX = Ovariectomy; RC = Red clover dry extracts; PCP = Pomegranate Concentrate Powder; RC:PCP = Mixed formulations consisted of RC and PCP (g/g); L = Lumen; MU = Mucosa; ML = Muscular layer; UG = Uterine gland. All H&E stain. Scale bars = 120 μm.

Significant decreases in total, mucosa, and epithelial thicknesses of the uterus, and in the percentages of uterine glands in the mucosa, were observed in OVX-induced mice due to estrogen depletion-related atrophic changes. However, total, mucosa, and epithelial thicknesses of the uterus, as well as in the percentages of uterine glands in the mucosain estradiol- and 1:1, 1:2, 1:4, 1:8, 2:1, 4:1, and 8:1 RC:PCP mixed formula-treated mice were the higher than in OVX control mice. Especially, significant normalization in OVX-induced uterine atrophic histopathological changes were detected in RC:PCP 2:1, 1:1 and 4:1 mixed formula treated mice compared with RC- or PCP-treated mice, in that orders, respectively (Table 5, Figure 3). 

**Figure 4:**
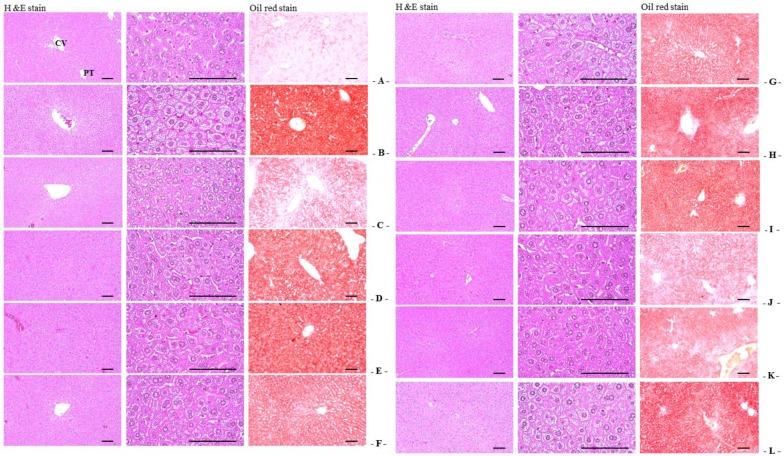
Representative histological images of the left lateral lobes of liver, taken from sham-operated or OVX ddY mice. (**A**) Sham vehicle control mice; (**B**) OVX control mice; (**C**) 17β-estradiol OVX induced mice; (**D**) RC alone administered OVX induced mice; (**E**) PCP alone administered OVX induced mice; (**F**–**L**) RC:PCP 1:1, 1:2, 1:4, 1:8, 2:1, 4:1, and 8:1 mixtures administered OVX-induced mice, respectively. OVX = Ovariectomy; RC = Red clover dry extracts; PCP = Pomegranate Concentrate Powder; RC:PCP = Mixed formulations consisted of RC and PCP (g/g); CV = Central vein; PT = Portal Triad. Scale bars = 120 μm.

Significant increases in the percentage of fatty change regions and mean diameters of hepatocytes were observed in OVX-induced mice due to the deposition of lipids into hepatocytes and steatosis. However, the percentage of fatty change regions and mean diameters of hepatocytes in all test substance-administered mice (including estradiol-treated mice) were the lower than in OVX control mice. Especially, RC:PCP 2:1, 1:1 and 4:1 mixed formula treated mice showed more significant inhibition in hepatic steatosis—increases of the percentage of fatty change regions and mean diameters of hepatocytes induced by OVX than in RC- or PCP-treated mice, in that orders, respectively (Table 4, Figure 4).

### 3.5. Femur Weight

Femur relative wet-weights and absolute and relative dry and ash weights were the lower in OVX-induced mice than in sham control mice. However, the femur wet relative weight and dry and ash absolute and relative weights in all test substance-treated mice, including estradiol treated mice, were the higher than in OVX control mice. Especially, RC:PCP (2:1, 1:1, and 4:1)-treated mice were higher than in OVX control mice (Table 6).

### 3.6. Femur Histopathology 

**Figure 5:**
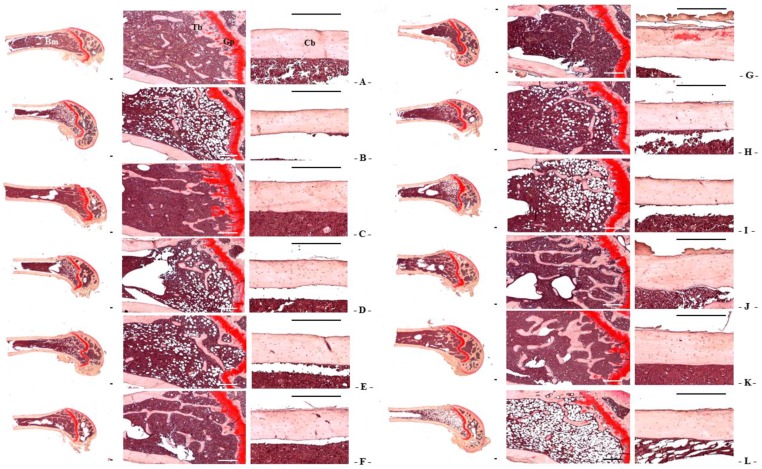
Representative histological profiles of the left femur, taken from sham-operated or OVX ddY mice. (**A**) Sham vehicle control mice; (**B**) OVX control mice; (**C**) 17β-estradiol OVX induced mice; (**D**) RC alone administered OVX induced mice; (**E**) PCP alone administered OVX induced mice; (**F**–**L**) RC:PCP 1:1, 1:2, 1:4, 1:8, 2:1, 4:1, and 8:1 mixtures administered OVX-induced mice, respectively. OVX = Ovariectomy; RC = Red clover dry extracts; PCP = Pomegranate Concentrate Powder; RC:PCP = Mixed formulations consisted of RC and PCP (g/g); Cb = cortical bone; Tb = trabecular bone; Bm = bone marrow; Gp = growth plate. All Safranin O stain. Scale bars = 240 μm.

Although relatively well-developed trabecular and cortical bone were observed in the femur of sham control mice, classical osteoporotic histological profiles were observed in OVX-induced mice as significant decreases in trabecular and cortical bone masses and increases in connective tissues in periosteum of cortical bone resulting from resorption of osteoid tissues related to osteoclast activation. However, bone mass and structures, of both trabecular and cortical bones in all test substance-administered mice were the higher than in OVX control mice, which is related to their inhibitory activities on osteoclast cell activities. Especially, RC:PCP 2:1, 1:1 and 4:1 mixed formula treated mice showed the lower noticeable inhibition in the bone losses and osteoclast cell activations than RC- or PCP-treated mice, in that orders, respectively (Table 7 and Table 8, Figure 5).

TV/BV, Tbn, Tbt, Tbl, and Cbt were the higher in OVX-induced mice than in sham-operated control mice in the femur. However, bone mass and structures in estradiol and 1:1, 1:2, 1:4, 1:8, 2:1, 4:1, and 8:1 RC:PCP mixed formulae—treated mice were the lower than in OVX control mice, respectively (Table 7 and Table 8, Figure 5). Ocn and OS/BS were the higher in OVX-induced mice than in sham control mice, in the femur. However, these activations and increases in osteoclast cells were in all test substances including estradiol were the lower than in OVX control mice. Especially, RC:PCP 2:1, 1:1 and 4:1 mixed formula treated mice showed the lower in the inhibitory activity against bone mass depletion and destroy of structure induced by OVX than RC- or PCP-treated mice, in that orders, respectively (Table 8, Figure 5).

### 3.7. Biochemical Variable

**Figure 6:**
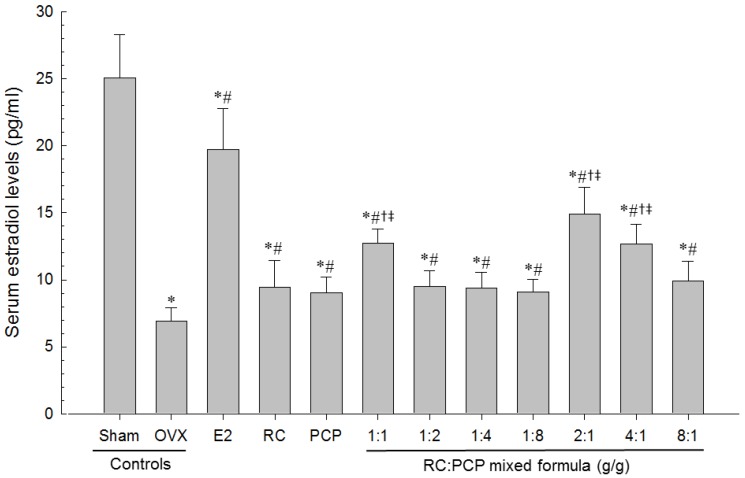
Serum Estradiol Levels in Sham-operated or OVX ddY Mice. Values are expressed mean ± S.D. of eight mice. OVX = Ovariectomy; RC = Red clover dry extracts; PCP = Pomegranate Concentrate Powder; RC:PCP = Mixed formulations consisted of RC and PCP (g/g). * *p* < 0.05 as compared with sham control; ^#^ *p* < 0.05 as compared with OVX control; ^†^ *p* < 0.05 as compared with RC single formula treated mice; ^‡^ *p* < 0.05 as compared with PCP single formula treated mice.

**Figure 7:**
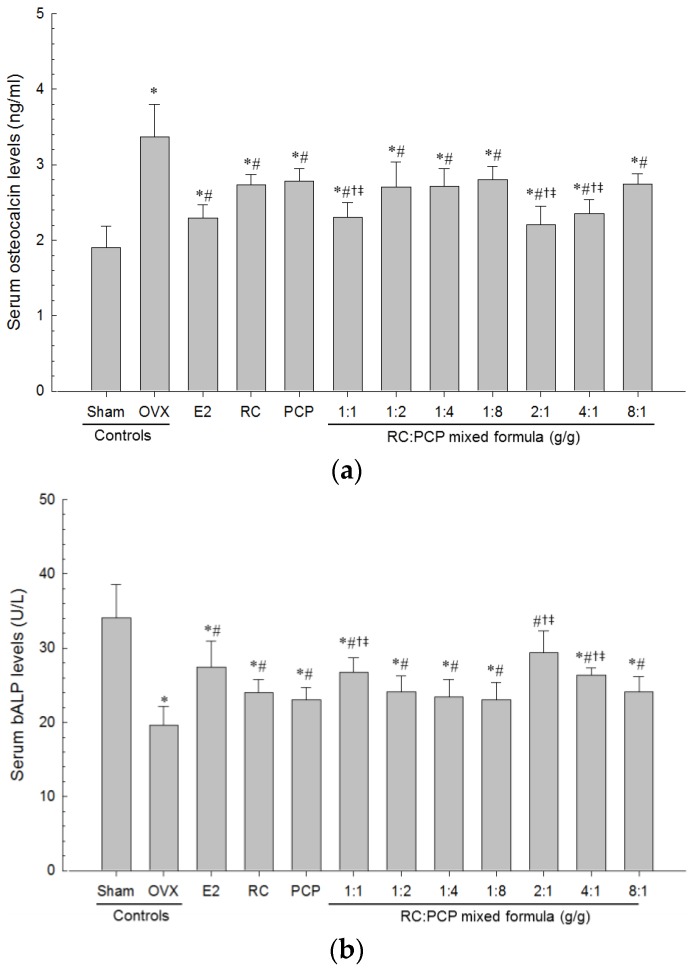
Serum (**a**) Osteocalcin and (**b**) bALP Levels in Sham-operated or OVX ddY Mice. Values are expressed mean ± S.D. of eight mice. OVX = Ovariectomy; E2 = 17β-estradiol; RC = Red clover dry extracts; PCP = Pomegranate Concentrate Powder; RC:PCP = Mixed formulations consisted of RC and PCP (g/g). * *p* < 0.05 as compared with sham control; ^#^ *p* < 0.05 as compared with OVX control; ^†^ *p* < 0.05 as compared with RC single formula treated mice; ^‡^ *p* < 0.05 as compared with PCP single formula treated mice.

Serum AST, ALT, TC, LDL, TG and osteocalcin levels were higher and serum HDL, estradiol and bALP levels were the lower in OVX-induced mice than in sham control mice. However, serum AST, ALT, TC, LDL, TG, and osteocalcin levels and serum HDL, estradiol and bALP levels in all test material-treated mice were the lower and higher than in OVX control mice, respectively. Especially, RC:PCP 2:1, 1:1 and 4:1 mixed formula treated mice showed the lower than in single formula of RC- or PCP-treated mice, in that orders, respectively (Table 9, Figure 6 and Figure 7).

### 3.8. Bone Mineral and Body Fat Density

Total body and femur mean BMDs were the lower and total body and abdominal fat densities were the higher in OVX-induced mice than in sham control mice. However, total body and femur mean BMD in estradiol- and all test material-administered mice were the higher than in OVX control mice, otherwise total body and abdominal fat densities were the lower in all material treated mice than OVX control mice. Especially, the RC:PCP 2:1, 1:1, and 4:1 mixtures showed the higher BMD and the lower fat densities than in RC or PCP alone-treated mice (Table 10).

### 3.9. Effects of RC/PCP Mixture on Bone Strength

**Figure 8:**
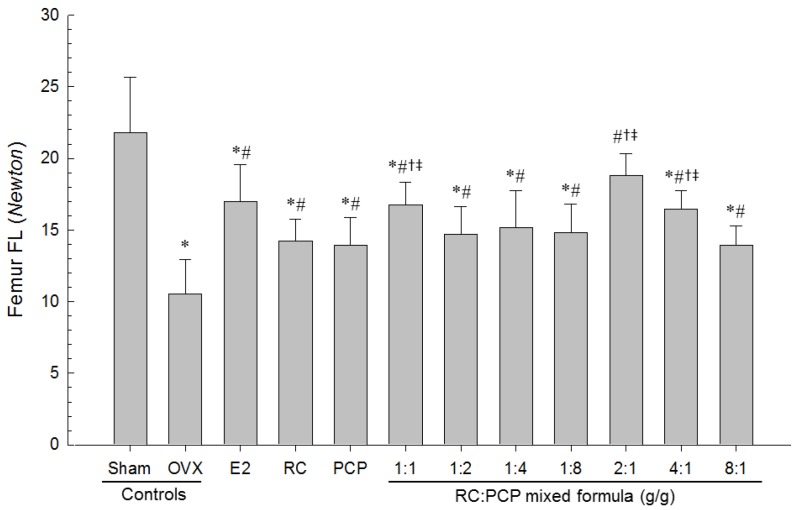
Femur FL in Sham-operated or OVX ddY mice. Values are expressed mean ± S.D. of eight mice. OVX = Ovariectomy; E2 = 17β-estradiol; RC = Red clover dry extracts; PCP = Pomegranate Concentrate Powder; RC:PCP = Mixed formulations consisted of RC and PCP (g/g); FL = Failure load, bone strength. * *p* < 0.05 as compared with sham control; ^#^ *p* < 0.05 as compared with OVX control; ^†^ *p* < 0.05 as compared with RC single formula treated mice; ^‡^ *p* < 0.05 as compared with PCP single formula treated mice.

The strengths (FL) of femur mid-shaft regions in OVX-induced mice were the lower than in sham control mice, but FL on the femur in all test substance-administrated mice including 1 mL/kg PCP-treated mice were the higher than in OVX control mice. Especially, RC:PCP 2:1, 1:1, and 4:1 mixtures showed the lower in FL in the femur mid-shaft regions than in RC- or PCP-treated mice, in that orders, respectively (Figure 8).

## 4. Discussion

In this study, we found that RC with PCP alleviated climacteric symptoms, such as obesity, hyperlipidemia, hepatic steatosis, and osteoporosis, in OVX mice. Our results indicated that RC:PCP mixed formulae (RC:PCP 2:1, 1:1, and 4:1) exerted enhanced pharmacological effects, compared with RC or PCP alone treatment. Large evidence suggested that pomegranate juice and pomegranate polyphenol extracts can protect women from many types of cancer, cardiovascular disease, diabetes, Alzheimer’s disease, arthritis, and colitis [25,36,37]. In addition, pomegranate seed oil and pomegranate juice including flavonoids and anthocyanidins possessed a strong antioxidant activity when compared to that in red wine or green tea extract [38,39]. Flavonoids can interact directly with estrogen receptors and regulate the activity of CYP19, which catalyzes the rate-limiting step in estrogen biosynthesis [40]. These property results in alterations of the overall hormonal balance, consequently leading to prevent bone loss and reducing osteoporotic effects and other menopausal symptoms [41]. These evidences support that the favorable effects of RC [14,22,23] and PCP [26,39] against OVX-induced symptoms are mainly associated with the antioxidant effects of isoflavonoids [15,27]. Therefore, appropriate mixtures consisting of RC and PCP may show greater protective effects by combination of isoflavonoids and ellagic acid [42,43]. The RC and PCP mixture may enhance direct free radical scavenging and indirectly induce antioxidative enzymes. It was thus assumed that appropriate RC:PCP mixtures may show more favorable synergistic anti-climacteric effects. Previously, no information to determine the optimal ranges of RC with PCP existed. Our results suggest that among the seven RC:PCP mixed formulae tested, RC:PCP 2:1 mixture showed the greatest inhibition against the climacteric symptoms induced by OVX in this experiment. Thus, our findings are interesting in that PCP exerted a potentially synergistic effect with other antioxidants, such as RC.

OVX induced marked increases in food consumption, body weight and gains, and abdominal fat deposition with adipocyte hypertrophy, which were inhibited significantly by 2:1, 1:1, and 4;1 RC:PCP mixed formulae. The RC:PCP 2:1 mixture showed the strongest inhibitory activities against obesity induced by OVX. Estrogen deficiency induced by OVX markedly accelerated food intake and changes in body fat deposition in the abdominal cavity. Additionally, an accumulation of fat deposition and cellular hypertrophy through expansion of intra-abdominal adipose tissue were observed in obesity-induced OVX mice. The correlation between estradiol and cholecystokinin (CCK) is well documented [44,45]. In addition, OVX models have been used to explain the mechanism of the operation of glucagon, because the activities of glucagon and glucagon antibodies on reduced and augmented meal sizes were both enhanced by estradiol [46]. Estradiol has been regarded as a modulator of eating and body weight by regulating the potency of the feedback signals that control meal size [46,47]. Under estradiol deficiency, eating and body weight are increased [48,49,50]. There is evidence of clinical relevance in that postmenopausal women show decreased estradiol levels; also, a high proportion of the obese population is represented by postmenopausal women [51]. It is assumed that the anti-obesity effects of the RC:PCP mixture may be related to estrogenic food intake effects, but more complex mechanisms are involved in the anti-obesity effects of PCP. Generally, enhanced digestive motility increases in fecal excretion, leading to a reduction in body weight in rodents [52,53].

OVX-induced mice showed a significant increase in serum TC, LDL, and TG levels, but decreased serum HDL contents. However, the hyperlipidemia resulting from OVX was inhibited significantly by RC:PCP 2:1, 1:1, and 4:1 mixed formulae. This finding is similar to previous reports showing a significant increase in TC, LDL, and TG, and low HDL levels, in postmenopausal women [54]; similar trends in serum lipids were observed in OVX induced mice [55]. The effects of estradiol on serum lipid profiles are believed to be mediated by inhibiting the activity of 3-hydroxy-3-methylglutaryl coenzyme A reductase (HMG-CoA) [56]. Because HMG-CoA is the rate-limiting enzyme involved in cholesterol synthesis, these effects may occur through the elevation of HMG-CoA activity, which is associated with cholesterol synthesis [56]. 

OVX-induced liver steatosis was observed, while 2:1, 1:1, and 4:1 RC:PCP mixed formulae inhibited OVX-induced hepatic steatosis significantly. As the liver is the major target organ of HMG-CoA reductase [34,57], AST and ALT activities were increased, and hypertrophy and fatty changes were observed in hepatocytes [58,59]. These are related to estrogen deficiency-mediated obesity and hyperlipidemia [34,59,60]. Estrogen deficiency is attributed to an atherogenic lipid profile, characterized by HDL-cholesterol, LDL-cholesterol, triglyceride levels [5], central adiposity [6], increased diastolic pressure [7], and increased insulin resistance [8].

Estrogen-deficiency osteoporosis induced by bilateral OVX was markedly inhibited by RC and PCP single formulae and by the RC:PCP 2:1, 1:1, 4:1 mixed formulae. These effects are related with the increased diversity of isoflavonoids in the mixtures, at least, under these experimental conditions. Previous studies reported that each single formula of RC [23,61] or PCP [26,39] has anti-osteoporotic effects. Bone loss is aggravated in menopausal women with aging, because of the loss of estrogen. Osteoporosis is a bone disease that occurs due to an imbalance between bone resorption and bone formation, which results in bone loss and structural deterioration of bone [62]. When determining anti-osteoporotic activities, the increase in bone weight is used as a good marker; however, it is difficult to evaluate anti-osteoporotic agents through changes in bone weight, with the exception of ash bone weight [63]. For an osteoporosis-related OVX model, bone turnover markers, including serum bALP content and osteocalcin levels, are appropriate [64,65,66], and BMD is considered a pivotal determinant of osteoporosis [67,68,69]. The observation of bone morphology using microscopy is good for evaluating anti-osteoporotic agents [31,35,70,71], especially, the trabecular and cortical bone changes significant in osteoporotic animals. In addition, some histomorphometric indices of bone mass and bone formation are decreased markedly, whereas histomorphometric indices of bone resorption are increased [31,35,72]. Thus, to evaluate the efficacy of various anti-osteoporosis agents, the histology of bones has been regard as an appropriate approach [31,35,71]. The RC:PCP mixed formulae evaluated here showed anti-osteoporotic activities similar to previous results [26,39].

OVX-induced uterine weight along with marked decreases in serum estradiol levels and related uterine atrophic changes, i.e., decreases in total, mucosa and epithelial thicknesses, and uterine glands in the mucosa were suppressed significantly by the RC:PCP mixed formulae. Loss of estrogen after menopause in women leads to climacteric symptoms [73,74], because estrogens affect numerous female target organs, such as the uterus, vagina, and skeletal and cardiovascular systems [75,76]. Recent study reported that soy isoflavone supplementation has no influence in endometrial thickness or in the rates of endometrial hyperplasia and female cancer in postmenopausal women [24]. In contrast, the other study reported that genistein (0.7 mg per day) had protective effect on trabecular bone loss in OVX control mice without hypertrophic effects on the uterus, while administration of genistein (5 mg per day) resulted in uterine hypertrophy. These data indicate that there is a marked difference between genistein dosages that prevent bone loss and those that induce uterine hypertrophy [77]. Genestein and specific ERβ agonist consumption may prevent the ileal and colonic epithelium from tumor development via modulation of tissue homeostasis [78]. Genistein enhanced the basal Toll like receptor 2 (TLR2) and reduced the viral component-induced TLR2 protein expression in human endometrial epithelial cells may suggest the potential role of this soy isoflavone in stimulating the uterine immune function and probably attenuating the inflammation of endometrium following pathogen [79]. Isoflavonoids increase in uterine masses via uterine water imbibition and/or cell proliferation [75,80], which are mediated by ERα [81,82,83]. However, there had limitation in this study that the increase of estradiol level was not explained clearly, more detail mechanism should be studied in future.

## 5. Conclusions

Taken together, our results indicate that an appropriate mixed formula consisting of RC and PCP (RC:PCP 2:1, 1:1, and 4:1 mixtures) synergistically increased the anti-climacteric effects—estrogenic, antiobesity, hypolipidemic, hepatoprotective, and antiosteoporotic effects—of RC- or PCP-alone in OVX induced mice. The RC:PCP 2:1 mixture, specifically, showed the most favorable inhibitory activities against estrogen-deficiency climacteric symptoms induced by OVX in this study. It is thus expected that the RC:PCP 2:1 mixture may have promise as a new potent protective agent for relieving climacteric symptoms, especially in terms of estrogen depletion, obesity, hyperlipidemia, hepatic steatosis, and osteoporosis, in menopausal women.

## Figures and Tables

**Table 1:** Ingredient of diets used in this study.

Ingredient (g/kg Diet)	
Casein	200
l-Cystein	3
Corn starch	150
Sucrose	500
Cellulose	50
Soybean oil	50
Lard	0
Mineral mixture	35
Vitamin mixture	10
Choline bitartrate	2
Energy (kcal/g)	4.00
Protein (% kcal)	20
Carbohydrate (% kcal)	64
Fat (% kcal)	16

**Table 2:** Body weight gains in Sham-operated or OVX ddY Mice.

Periods Groups	Body Weights (g)			Body Weight Gains During Treatment (g)
	At OVX ^a^	At Initial Treatment ^a^	At Sacrifice ^a^	
Controls				
Sham	23.61 ± 1.64	24.75 ± 1.81	28.00 ± 2.23	3.25 ± 1.92
OVX	24.21 ± 1.47	30.78 ± 2.12 *	42.90 ± 2.28 *	14.68 ± 2.88 *
Estradiol	24.65 ± 0.99	28.71 ± 1.61 *	35.68 ± 2.19 *^,#^	6.96 ± 1.21 *^,#^
RC	23.93 ± 1.47	28.98 ± 1.73 *	36.44 ± 1.52 *^,#^	8.15 ± 0.95 *^,#^
PCP	24.60 ± 1.19	28.78 ± 2.02 *	37.30 ± 2.38 *^,#^	8.53 ± 1.31 *^,#^
RC:PCP				
1:1	24.41 ± 0.97	28.53 ± 1.65 *	33.26 ± 1.85 *^,#,†,‡^	4.74 ± 1.55 ^#,†,‡^
1:2	24.69 ± 1.33	28.56 ± 1.78 *	35.24 ± 2.54 *^,#^	6.68 ± 2.55 *^,#^
1:4	24.40 ± 0.97	28.44 ± 1.52 *	35.59 ± 2.05 *^,#^	7.15 ± 1.86 *^,#^
1:8	24.66 ± 1.11	28.69 ± 1.31 *	35.69 ± 1.95 *^,#^	7.00 ± 2.58 *^,#^
2:1	24.58 ± 1.08	28.50 ± 1.54 *	32.29 ± 2.53 *^,#,†,‡^	3.79 ± 2.28 ^#,†,‡^
4:1	24.31 ± 1.86	28.41 ± 0.88 *	33.64 ± 1.45 *^,#,†,‡^	5.23 ± 1.80 ^#,†,‡^
8:1	24.61 ± 1.77	28.73 ± 1.12 *	36.86 ± 4.74 *^,#^	8.14 ± 4.99 *^,#^

Values are expressed mean ± S.D. of eight mice. ^a^ All animals were overnight fasted; * *p* < 0.01 as compared with sham control; ^#^ *p* < 0.05 as compared with OVX control; ^†^ *p* < 0.05 as compared with RC single formula treated mice; ^‡^ *p* < 0.01 as compared with PCP single formula treated mice; OVX = Bilateral ovariectomy; RC = Red clover dry extracts; PCP = Pomegranate Concentrate Powder; RC:PCP = Mixed formulations consisted of RC and PCP (g/g).

**Table 3:** Abdominal fat pad, uterus and liver weights in sham-operated or OVX ddY mice.

Organs Groups	Relative Wet-Weight (% of Body Weight)		
	Abdominal Fat Pad	Uterus	Liver
Controls			
Sham	0.492 ± 0.390	0.858 ± 0.286	4.291 ± 0.819
OVX	7.835 ± 0.814 *	0.064 ± 0.022 *	2.929 ± 0.369 *
Estradiol	1.656 ± 1.432 *^,#^	0.303 ± 0.124 *^,#^	3.724 ± 0.307 ^#^
RC	4.563 ± 1.417 *^,#^	0.112 ± 0.018 *^,#^	3.571 ± 0.429 ^#^
PCP	4.970 ± 1.163 *^,#^	0.107 ± 0.013 *^,#^	3.552 ± 0.388 ^#^
RC:PCP			
1:1	0.157 ± 0.010 *^,#,†,‡^	4.166 ± 0.165 ^#,†,‡^	1.386 ± 0.095
1:2	0.118 ± 0.022 *^,#^	3.508 ± 0.525 *^,#^	1.234 ± 0.192
1:4	0.111 ± 0.020 *^,#^	3.549 ± 0.576 ^#^	1.254 ± 0.140
1:8	0.111 ± 0.014 *^,#^	3.590 ± 0.487 ^#^	1.275 ± 0.131
2:1	0.193 ± 0.032 *^,#,†,‡^	4.392 ± 0.428 ^#,†,‡^	1.411 ± 0.092 ^†^
4:1	0.155 ± 0.028 *^,#,†,‡^	4.174 ± 0.286 ^#,†,‡^	1.404 ± 0.107 ^†^
8:1	0.116 ± 0.029 *^,#^	3.456 ± 0.352 *^,#^	1.263 ± 0.103

Values are expressed mean ± S.D. of eight mice. * *p* < 0.05 as compared with sham control; ^#^ *p* < 0.05 as compared with OVX control; ^†^ *p* < 0.05 as compared with RC single formula treated mice; ^‡^ *p* < 0.05 as compared with PCP single formula treated mice; OVX = Bilateral ovariectomy; RC = Red clover dry extracts; PCP = Pomegranate Concentrate Powder; RC:PCP = Mixed formulations consisted of RC and PCP (g/g).

**Table 4:** Histopathology-Histomorphometry for the Abdominal Fat Pads and Liver in Sham-operated or OVX ddY Mice.

Items Groups	Abdominal Fat Pads		Hepatic Tissues	
	Total Thickness (μm)	Mean Adipocyte Diameters (μm)	Steatosis Regions (%)	Mean Hepatocyte Diameters (μm)
Controls				
Sham	1.41 ± 0.45	35.05 ± 10.25	13.08 ± 3.16	9.83 ± 3.34
OVX	6.04 ± 0.75 *	129.93 ± 24.06 *	78.58 ± 6.76 *	31.93 ± 4.92 *
Estradiol	2.89 ± 0.27 *^,#^	67.49 ± 14.77 *^,#^	43.43 ± 6.53 *^,#^	18.45 ± 3.66 *^,#^
RC	4.48 ± 0.51 *^,#^	92.85 ± 12.70 *^,#^	59.42 ± 7.08 *^,#^	23.47 ± 3.83 *^,#^
PCP	4.70 ± 0.63 *^,#^	97.27 ± 15.68 *^,#^	62.01 ± 7.82 *^,#^	23.66 ± 3.59 *^,#^
RC:PCP				
1:1	3.58 ± 0.49 *^,#,†,‡^	55.57 ± 12.55 *^,#,†,‡^	44.63 ± 6.94 *^,#,†,‡^	16.00 ± 3.73 *^,#,†,‡^
1:2	4.59 ± 0.45 *^,#^	88.91 ± 15.64 *^,#^	58.37 ± 7.54 *^,#^	22.28 ± 4.15 *^,#^
1:4	4.55 ± 0.65 *^,#^	89.01 ± 13.89 *^,#^	59.80 ± 6.51 *^,#^	22.34 ± 2.75 *^,#^
1:8	4.67 ± 0.61 *^,#^	95.77 ± 18.09 *^,#^	61.13 ± 10.91 *^,#^	23.83 ± 2.67 *^,#^
2:1	2.71 ± 0.93 *^,#,†,‡^	47.17 ± 10.84 ^#,†,‡^	37.84 ± 9.05 *^,#,†,‡^	14.24 ± 4.07 *^,#,†,‡^
4:1	3.69 ± 0.50 *^,#,†,‡^	62.87 ± 14.30 *^,#,†,‡^	45.99 ± 8.44 *^,#,†,‡^	17.53 ± 3.43 *^,#,†,‡^
8:1	4.47 ± 0.75 *^,#^	94.54 ± 14.59 *^,#^	59.98 ± 7.88 *^,#^	23.41 ± 4.31 *^,#^

Values are expressed mean ± S.D. of eight mice. * *p* < 0.05 as compared with sham control; ^#^ *p* < 0.01 as compared with OVX control; ^†^ *p* < 0.05 as compared with RC single formula treated mice; ^‡^ *p* < 0.01 as compared with PCP single formula treated mice; OVX = Bilateral ovariectomy; RC = Red clover dry extracts; PCP = Pomegranate Concentrate Powder; RC:PCP = Mixed formulations consisted of RC and PCP (g/g).

**Table 5:** Histopathology-Histomorphometry for the uterus in sham-operated or OVX ddY mice.

Items Groups	Left Uterine Horn Tissues			
	Total Thickness (μm)	Epithelium Thickness (μm)	Mucosa Layer Thickness (μm)	Uterine Gland Regions (%)
Controls				
Sham	2177.82 ± 573.19	34.58 ± 6.17	951.22 ± 242.86	53.94 ± 12.02
OVX	566.47 ± 128.78 *	7.68 ± 1.26 *	202.25 ± 46.58 *	12.46 ± 3.07 *
Estradiol	1593.32 ± 371.85 *^,#^	20.75 ± 4.52 *^,#^	598.74 ± 153.51 *^,#^	34.93 ± 4.60 *^,#^
RC	776.35 ± 97.07 *^,#^	11.62 ± 3.02 *^,#^	295.40 ± 45.06 *^,#^	24.03 ± 6.13 *^,#^
PCP	739.04 ± 84.66 *^,#^	11.06 ± 2.72 *^,#^	282.10 ± 37.26 *^,#^	22.47 ± 4.28 *^,#^
RC:PCP				
1:1	1003.98 ± 173.11 *^,#,†,‡^	17.96 ± 4.26 *^,#,†,‡^	409.30 ± 31.93 *^,#,†,‡^	32.91 ± 3.70 *^,#,†,‡^
1:2	789.85 ± 116.09 *^,#^	13.16 ± 4.17 *^,#^	314.95 ± 79.42 *^,#^	26.10 ± 6.70 *^,#^
1:4	776.76 ± 98.02 *^,#^	12.46 ± 3.50 *^,#^	297.63 ± 45.24 *^,#^	23.88 ± 3.85 *^,#^
1:8	746.54 ± 64.96 *^,#^	11.22 ± 2.41 *^,#^	285.57 ± 40.39 *^,#^	22.54 ± 4.56 *^,#^
2:1	1124.93 ± 125.59 *^,#,†,‡^	23.46 ± 4.93 *^,#,†,‡^	455.64 ± 92.39 *^,#,†,‡^	35.65 ± 6.07 *^,#,†,‡^
4:1	917.42 ± 54.64 *^,#,†,‡^	16.87 ± 4.11 *^,#,†,‡^	372.29 ± 44.26 *^,#,†,‡^	32.37 ± 4.67 *^,#,†,‡^
8:1	796.67 ± 146.28 *^,#^	12.11 ± 3.99 *^,#^	309.44 ± 44.82 *^,#^	24.71 ± 4.22 *^,#^

Values are expressed mean ± S.D. of eight mice. * *p* < 0.05 as compared with sham control; ^#^ *p* < 0.05 as compared with OVX control; ^†^ *p* < 0.05 as compared with RC single formula treated mice; ^‡^ *p* < 0.01 as compared with PCP single formula treated mice. OVX = Bilateral ovariectomy; RC = Red clover dry extracts; PCP = Pomegranate Concentrate Powder; RC:PCP = Mixed formulations consisted of RC and PCP (g/g).

**Table 6:** Right femur weights in sham-operated or OVX ddY mice.

Items Groups	Absolute Weight (g)			Relative Weight (% of Body Weight)		
	Wet	Dry	Ash	Wet	Dry	Ash
Controls						
Sham	0.092 ± 0.005	0.064 ± 0.003	0.039 ± 0.003	0.331 ± 0.028	0.213 ± 0.025	0.139 ± 0.019
OVX	0.089 ± 0.006	0.050 ± 0.003 *	0.025 ± 0.003 *	0.207 ± 0.013 *	0.118 ± 0.010 *	0.059 ± 0.007 *
Estradiol	0.094 ± 0.007	0.058 ± 0.004 *^,#^	0.033 ± 0.002 *^,#^	0.264 ± 0.032 *^,#^	0.162 ± 0.016 *^,#^	0.092 ± 0.007 *^,#^
RC	0.090 ± 0.004	0.055 ± 0.002 *^,#^	0.031 ± 0.003 *^,#^	0.246 ± 0.015 *^,#^	0.151 ± 0.011 *^,#^	0.084 ± 0.010 *^,#^
PCP	0.091 ± 0.005	0.055 ± 0.002 *^,#^	0.030 ± 0.003 *^,#^	0.244 ± 0.019 *^,#^	0.147 ± 0.010 *^,#^	0.081 ± 0.008 *^,#^
RC:PCP						
1:1	0.092 ± 0.003	0.059 ± 0.003 *^,#,†,‡^	0.035 ± 0.001 *^,#,†,‡^	0.277 ± 0.017 *^,#,†,‡^	0.177 ± 0.006 *^,#,†,‡^	0.105 ± 0.006 *^,#,†,‡^
1:2	0.089 ± 0.005	0.056 ± 0.004 *^,#^	0.031 ± 0.004 *^,#^	0.254 ± 0.021 *^,#^	0.159 ± 0.018 *^,#^	0.088 ± 0.009 *^,#^
1:4	0.089 ± 0.005	0.056 ± 0.003 *^,#^	0.032 ± 0.004 *^,#^	0.251 ± 0.022 *^,#^	0.156 ± 0.014 *^,#^	0.089 ± 0.016 *^,#^
1:8	0.088 ± 0.007	0.055 ± 0.005 *	0.031 ± 0.004 *^,#^	0.247 ± 0.021 *^,#^	0.155 ± 0.015 *^,#^	0.086 ± 0.011 *^,#^
2:1	0.093 ± 0.003	0.061 ± 0.002 *^,#,†,‡^	0.038 ± 0.002 ^#,†,‡^	0.289 ± 0.021 *^,#,†,‡^	0.189 ± 0.016 *^,#,†,‡^	0.118 ± 0.012 *^,#,†,‡^
4:1	0.092 ± 0.005	0.059 ± 0.003 *^,#,†,‡^	0.034 ± 0.003 *^,#,†,‡^	0.272 ± 0.016 *^,#,†,‡^	0.175 ± 0.012 *^,#,†,‡^	0.102 ± 0.010 *^,#,†,‡^
8:1	0.089 ± 0.007	0.055 ± 0.005 *	0.031 ± 0.003 *^,#^	0.245 ± 0.029 *^,#^	0.152 ± 0.021 *^,#^	0.084 ± 0.014 *^,#^

Values are expressed mean ± S.D. of eight mice. * *p* < 0.05 as compared with sham control; ^#^ *p* < 0.05 as compared with OVX control; ^†^ *p* < 0.05 as compared with RC single formula treated mice; ^‡^ *p* < 0.05 as compared with PCP single formula treated mice. OVX = Bilateral ovariectomy; RC = Red clover dry extracts; PCP = Pomegranate Concentrate Powder; RC:PCP = Mixed formulations consisted of RC and PCP (g/g).

**Table 7:** Histopathology-Histomorphometry for the femur in sham-operated or OVX ddY mice: trabecular bones.

Items Groups	Left Femur Tissues			
	TV/BV	Tbn	Tbl	Tbt
Controls				
Sham	37.28 ± 5.04	12.63 ± 1.69	1029.36 ± 145.55	73.44 ± 11.98
OVX	17.28 ± 2.91 *	5.13 ± 0.64 *	478.99 ± 80.94 *	32.45 ± 7.07 *
Estradiol	29.89 ± 5.24 *^,#^	9.88 ± 1.36 *^,#^	905.51 ± 146.34 ^#^	55.34 ± 8.99 *^,#^
RC	22.89 ± 2.05 *^,#^	7.50 ± 0.93 *^,#^	592.13 ± 35.08 *^,#^	44.03 ± 3.97 *^,#^
PCP	21.69 ± 1.85 *^,#^	7.00 ± 1.20 *^,#^	578.99 ± 39.41 *^,#^	43.25 ± 2.49 *^,#^
RC:PCP				
1:1	27.63 ± 2.47 *^,#,†,‡^	10.38 ± 1.30 *^,#,†,‡^	859.44 ± 173.38 *^,#,†,‡^	66.28 ± 14.40 ^#,†,‡^
1:2	22.65 ± 2.58 *^,#^	7.50 ± 0.76 *^,#^	610.56 ± 60.98 *^,#^	46.85 ± 8.26 *^,#^
1:4	22.55 ± 2.72 *^,#^	7.38 ± 0.92 *^,#^	606.29 ± 36.21 *^,#^	46.62 ± 8.71 *^,#^
1:8	21.84 ± 1.57 *^,#^	6.88 ± 0.83 *^,#^	580.37 ± 66.93 *^,#^	43.80 ± 5.38 *^,#^
2:1	31.40 ± 4.14 *^,#,†,‡^	12.25 ± 1.04 *^,#,†,‡^	954.18 ± 140.48 ^#,†,‡^	74.33 ± 14.63 ^#,†,‡^
4:1	27.39 ± 3.68 *^,#,†,‡^	10.13 ± 1.89 *^,#,†,‡^	827.11 ± 142.28 *^,#,†,‡^	65.30 ± 11.84 ^#,†,‡^
8:1	22.59 ± 3.67 *^,#^	7.50 ± 0.93 *^,#^	594.17 ± 49.46 *^,#^	44.64 ± 3.53 *^,#^

Values are expressed mean ± S.D. of eight mice. * *p* < 0.05 as compared with sham control; ^#^ *p* < 0.05 as compared with OVX control; ^†^ *p* < 0.01 as compared with RC single formula treated mice; ^‡^ *p* < 0.01 as compared with PCP single formula treated mice. OVX = Bilateral ovariectomy; RC = Red clover dry extracts; PCP = Pomegranate Concentrate Powder; RC:PCP = Mixed formulations consisted of RC and PCP (g/g); TV/BV = Trabecular bone volume (%); Tbn = Trabecular bone number (N/epiphyseal); Tbl = Trabecular bone length (Longitudinal thickness; mm); Tbt = Trabecular bone thickness (Cross thickness; μm).

**Table 8:** Histopathology-Histomorphometry for the femur in sham-operated or OVX ddY mice: cortical bones and osteoclast cells.

Items Groups	Left Femur Tissues		
	Cbt	Ocn	OS/BS
Controls			
Sham	209.33 ± 18.14	5.50 ± 0.93	8.52 ± 1.57
OVX	151.14 ± 11.14 *	17.00 ± 2.00 *	21.00 ± 2.39 *
Estradiol	184.17 ± 8.40 *^,#^	7.63 ± 1.06 *^,#^	13.12 ± 1.41 *^,#^
RC	175.49 ± 4.41 *^,#^	13.75 ± 1.49 *^,†^	17.16 ± 1.89 *^,#^
PCP	172.05 ± 8.17 *^,#^	14.13 ± 1.64 *^,†^	17.75 ± 1.46 *^,#^
RC:PCP			
1:1	189.27 ± 7.10 *^,#,†,‡^	9.13 ± 1.13 *^,#,†,‡^	13.46 ± 1.88 *^,#,†,‡^
1:2	179.81 ± 8.89 *^,#^	13.00 ± 1.85 *^,#^	16.63 ± 1.74 *^,#^
1:4	176.39 ± 9.83 *^,#^	13.50 ± 1.51 *^,#^	17.08 ± 1.65 *^,#^
1:8	173.84 ± 9.20 *^,#^	13.88 ± 1.89 *^,#^	16.77 ± 3.23 *^,#^
2:1	197.13 ± 9.00 ^#,†,‡^	7.00 ± 0.76 ^#,†,‡^	11.46 ± 1.37 *^,#,†,‡^
4:1	187.92 ± 10.08 *^,#,†,‡^	9.38 ± 1.30 *^,#,†,‡^	13.64 ± 1.99 *^,#,†,‡^
8:1	172.30 ± 6.68 *^,#^	13.63 ± 2.33 *^,#^	17.82 ± 0.98 *^,#^

Values are expressed mean ± S.D. of eight mice. * *p* < 0.05 as compared with sham control; ^#^ *p* < 0.05 as compared with OVX control; ^†^ *p* < 0.01 as compared with RC single formula treated mice; ^‡^ *p* < 0.01 as compared with PCP single formula treated mice. OVX = Bilateral ovariectomy; RC = Red clover dry extracts; PCP = Pomegranate Concentrate Powder; RC:PCP = Mixed formulations consisted of RC and PCP (g/g); Cbt = Cortical bone thickness (Cross thickness; μm); Ocn = Osteoclast cell number (N/epiphyseal); OS/BS = Osteoclast cell surface/bone surface (%).

**Table 9:** Serum biochemistry: AST, ALT, TC, LDL, HDL and TG levels in sham-operated or OVX ddY mice.

Items Groups	Serum Biochemistrical Values					
	AST (U/L)	ALT (U/L)	TC (mg/dL)	LDL (mg/dL)	HDL (mg/dL)	TG (mg/dL)
Controls						
Sham	84.38 ± 13.95	37.88 ± 11.98	89.63 ± 16.87	62.88 ± 10.11	96.38 ± 11.78	37.00 ± 10.39
OVX	162.35 ± 15.84 *	76.00 ± 10.54 *	179.25 ± 21.41 *	181.88 ± 17.94 *	47.75 ± 11.20 *	150.13 ± 20.90 *
Estradiol	107.13 ± 12.43 *^,#^	52.00 ± 10.03 *^,#^	132.75 ± 21.06 *^,#^	133.00 ± 10.54 *^,#^	72.63 ± 11.04 *^,#^	97.88 ± 18.16 *^,#^
RC	139.63 ± 8.86 *^,#^	63.00 ± 5.66 *^,#^	143.38 ± 10.80 *^,#^	154.63 ± 13.05 *^,#^	65.50 ± 5.10 *^,#^	123.13 ± 9.11 *^,#^
PCP	141.88 ± 7.64 *^,#^	63.63 ± 4.84 *^,#^	147.25 ± 12.28 *^,#^	160.13 ± 7.41 *^,#^	63.63 ± 6.05 *^,#^	124.50 ± 9.38 *^,#^
RC:PCP						
1:1	118.88 ± 13.26 *^,#,†,‡^	51.88 ± 7.26 *^,#,†,‡^	127.50 ± 10.85 *^,#,†,‡^	133.00 ± 13.60 *^,#,†,‡^	6.88 ± 8.25 *^,#,†,‡^	102.75 ± 12.96 *^,#,†,‡^
1:2	139.38 ± 10.72 *^,#^	63.25 ± 7.46 *^,#^	142.25 ± 10.35 *^,#^	155.38 ± 16.23 *^,#^	65.38 ± 9.55 *^,#^	120.88 ± 14.80 *^,#^
1:4	140.00 ± 10.00 *^,#^	61.50 ± 11.33 *^,#^	143.50 ± 12.36 *^,#^	157.50 ± 11.63 *^,#^	63.50 ± 6.59 *^,#^	121.63 ± 11.59 *^,#^
1:8	142.50 ± 9.44 *^,#^	62.63 ± 6.72 *^,#^	147.88 ± 13.88 *^,#^	160.00 ± 9.96 *^,#^	64.00 ± 9.70 *^,#^	123.50 ± 12.14 *^,#^
2:1	109.25 ± 7.74 *^,#,†,‡^	7.75 ± 11.31 *^,#,†,‡^	114.13 ± 12.93 *^,#,†,‡^	115.50 ± 11.15 *^,#,†,‡^	89.63 ± 7.13 ^#,†,‡^	84.75 ± 13.64 ^#,†,‡^
4:1	120.13 ± 15.07 *^,#,†,‡^	52.88 ± 6.10 *^,#,†,‡^	131.13 ± 8.24 *^,#,†,‡^	138.75 ± 8.81 *^,#,†,‡^	76.13 ± 9.75 *^,#,†,‡^	107.25 ± 9.85 *^,#,†,‡^
8:1	141.00 ± 8.35 *^,#^	62.13 ± 11.01 *^,#^	144.38 ± 14.81 *^,#^	144.50 ± 12.24 *^,#^	64.13 ± 10.43 *^,#^	122.75 ± 9.68 *^,#^

Values are expressed mean ± S.D. of eight mice. * *p* < 0.05 as compared with sham control; ^#^ *p* < 0.01 as compared with OVX control; ^†^ *p* < 0.05 as compared with RC single formula treated mice; ^‡^ *p* < 0.05 as compared with PCP single formula treated mice. OVX = Bilateral ovariectomy; RC = Red clover dry extracts; PCP = Pomegranate Concentrate Powder; RC:PCP = Mixed formulations consisted of RC and PCP (g/g); ALT = Alanine aminotransferase; AST = Aspartate aminotransferase; LDL = Low density lipoprotein; TC = Total cholesterol; TG = Triglyceride; HDL = High density lipoprotein.

**Table 10:** Bone mineral density and body fat density in sham-operated or OVX ddY mice.

Items Groups	Bone Mineral Density (mg/cm^2^)		Fat Density (% of Body Mass)	
	Total Body	Right Femur	Total Body	Abdominal Cavity
Controls				
Sham	24.71 ± 0.59	26.78 ± 0.58	11.16 ± 2.15	10.85 ± 1.50
OVX	21.56 ± 0.30 *	23.41 ± 0.43 *	34.97 ± 3.52 *	41.15 ± 4.55 *
Estradiol	23.69 ± 0.44 *^,#^	25.94 ± 0.79 *^,#^	23.42 ± 4.24 *^,#^	26.75 ± 4.22 *^,#^
RC	22.51 ± 0.49 *^,#^	24.64 ± 0.45 *^,#^	29.65 ± 1.99 *^,#^	34.06 ± 4.04 *^,#^
PCP	22.31 ± 0.37 *^,#^	24.63 ± 0.32 *^,#^	30.35 ± 2.21 *^,#^	34.80 ± 2.81 *^,#^
RC:PCP				
1:1	23.29 ± 0.48 *^,#,†,‡^	25.57 ± 0.72 *^,#,†,‡^	24.11 ± 4.25 *^,#,†,‡^	26.62 ± 4.66 *^,#,†,‡^
1:2	22.66 ± 0.55 *^,#^	24.99 ± 1.09 *^,#^	27.92 ± 5.30 *^,#^	33.49 ± 4.25 *^,#^
1:4	22.49 ± 0.27 *^,#^	24.80 ± 0.67 *^,#^	29.49 ± 2.00 *^,#^	33.93 ± 4.26 *^,#^
1:8	22.38 ± 0.63 *^,#^	24.68 ± 0.90 *^,#^	30.10 ± 2.58 *^,#^	34.80 ± 3.48 *^,#^
2:1	23.90 ± 0.53 *^,#,†,‡^	26.11 ± 0.89 ^#,†,‡^	18.68 ± 3.99 *^,#,†,‡^	23.17 ± 3.39 *^,#,†,‡^
4:1	23.16 ± 0.37 *^,#,†,‡^	25.51 ± 0.70 *^,#,†,‡^	25.32 ± 3.08 *^,#,†,‡^	27.94 ± 3.74 *^,#,†,‡^
8:1	22.68 ± 0.82 *^,#^	24.59 ± 0.75 *^,#^	29.61 ± 2.64 *^,#^	33.88 ± 3.55 *^,#^

Values are expressed mean ± S.D. of eight mice. * *p* < 0.05 as compared with sham control; ^#^ *p* < 0.01 as compared with OVX control; ^†^ *p* < 0.05 as compared with RC single formula treated mice; ^‡^ *p* < 0.05 as compared with PCP single formula treated mice. OVX = Bilateral ovariectomy; RC = Red clover dry extracts; PCP = Pomegranate Concentrate Powder; RC:PCP = Mixed formulations consisted of RC and PCP (g/g).

## Appendix A. Body Weight Changes in Sham-Operated or OVX ddY Mice

Significant (*p* < 0.01) increases in body weight were detected in all OVX mice as compared with sham control mice, in this experiment (red arrow). However, significant decreases of body weights were demonstrated in estradiol, RC and PCP single formula treated mice from 14, 21 and 28 days after initial administration, and also from 14, 21 or 28 days after initial treatment in all nine types of RC:PCP 2:1 mixed formula (g/g) treated mice as compared with OVX control mice, respectively. In addition, all test substance treated mice showed significant decreases of body weight gains during 84 days of treatment periods as compared with OVX control (arrows). Especially, RC:PCP 2:1, 1:1 and 4:1 mixtures (g/g) treated OVX mice showed significant decreases of body weights as compared with each of single formula of RC and PCP treated mice from 28, 42 and 49 days after administration, respectively (dot arrows). Values are expressed mean ± S.D. of eight mice. OVX = Ovariectomy, RC = Red clover dry extracts, PCP = Pomegranate Concentrate Powder, RC:PCP = Mixed formulations consisted of RC and PCP (g/g). −1 means 1 day before start of administration at 27 days after OVX surgery, 0 means at start of administration, at 28 days after OVX, 84 means 84 days after start of administration, at sacrifice. All animals were overnight fasted before OVX, first administration and sacrifice, respectively.

## Appendix B. Food Consumptions in Sham-Operated or OVX ddY Mice

**Table:**

Points Groups	Food Consumption (g/24 h/Mouse): Days after Initial Treatment					
	1	3	7	28	56	83
Controls						
Sham	7.20 ± 0.72	6.35 ± 0.67	6.29 ± 0.72	6.59 ± 0.77	7.31 ± 0.90	7.54 ± 0.78
OVX	11.40 ± 1.44 *	11.11 ± 0.98 *	11.29 ± 1.03 *	11.38 ± 1.33 *	12.80 ± 1.41 *	14.75 ± 1.57 *
Estradiol	11.49 ± 1.41 *	10.53 ± 0.93 *	9.82 ± 0.93 *^,#^	9.52 ± 1.01 *^,#^	10.24 ± 0.98 *^,#^	10.65 ± 1.54 *^,#^
RC	11.26 ± 1.18 *	11.01 ± 1.53 *	11.06 ± 1.06 *	11.11 ± 0.92 *	12.66 ± 1.62 *	14.60 ± 1.08 *
PCP	11.91 ± 1.38 *	11.36 ± 1.67 *	11.52 ± 1.20 *	11.26 ± 1.31 *	12.87 ± 1.55 *	14.67 ± 1.34 *
RC:PCP						
1:1	11.46 ± 1.12 *	10.98 ± 1.34 *	11.13 ± 1.24 *	11.29 ± 1.31 *	12.43 ± 1.36 *	14.52 ± 1.40 *
1:2	11.08 ± 1.19 *	10.96 ± 1.25 *	11.33 ± 1.25 *	11.57 ± 1.19 *	13.04 ± 0.91 *	14.79 ± 1.35 *
1:4	11.24 ± 1.13 *	11.34 ± 1.11 *	11.58 ± 1.32 *	11.84 ± 1.44 *	12.45 ± 0.84 *	14.38 ± 1.68 *
1:8	11.62 ± 1.01 *	11.17 ± 1.40 *	11.37 ± 1.55 *	11.43 ± 1.35 *	12.95 ± 0.92 *	14.65 ± 2.04 *
2:1	11.66 ± 0.93 *	11.25 ± 0.95 *	11.04 ± 1.61 *	11.31 ± 1.14 *	12.90 ± 1.33 *	14.59 ± 1.62 *
4:1	11.42 ± 1.46 *	11.25 ± 0.99 *	11.40 ± 1.26 *	11.34 ± 1.26 *	12.76 ± 1.17 *	14.83 ± 1.23 *
8:1	11.34 ± 1.99 *	11.13 ± 1.13 *	11.37 ± 1.58 *	11.46 ± 1.25 *	12.86 ± 0.72 *	14.89 ± 1.17 *

Values are expressed mean ± S.D. of eight mice. * *p* < 0.05 as compared with sham control, ^#^ *p* < 0.05 as compared with OVX control. OVX = Bilateral ovariectomy, RC = Red clover dry extracts, PCP = Pomegranate Concentrate Powder, RC:PCP = Mixed formulations consisted of RC and PCP (g/g).
